# Mortality in the French Army in the East during the Late War

**Published:** 1856-10

**Authors:** 


### MISCELLANEOUS ITEMS.

*Mortality in the French Army in the East during the Late War.*

It has been our lot, on many occasions, to show that the chances of war are far less fatal to the soldier than the diseases he encounters when on foreign service. The sanitary history of the French Army of the East, during the late campaign in the Crimea, is full of facts which confirm the truth of this proposition in a manner so frightful and astounding that we have long doubted the accuracy of the accounts which have reached us from week to week; and it is only by the accumulated testimony of eye-witnesses, and the reports of medical officers high in the French service, that we have been able to admit the possibility of a rate of mortality among our allies so unprecedented as almost to exceed belief. For the truth of the following statements, however, we have the authority of medical officers both in our own and the French service, and have permission to name them if need be. They are not only interesting in themselves, but additionally so, as the facts have been studiously concealed by the French Government, and are now made known for the first time in this country: —

1. There were fourteen French hospitals in the Bosphorus up to the end of March. Since then three others have been added. The following is a copy of an official return of the patients treated in all the hospitals in January, February, and March, 1856: —

| | |
|---|---|
| January, - - - - - | 13,520 |
| February, - - - - - - | 21,309 |
| March, - - - - - - - | 28,167 |

2. During the ten days ending on the 20th of March, 1,009 *patients died;* and during the following ten days, 948 *patients died*, in these hospitals. The number of sick under treatment for all diseases on the 20th of March, was 11,366, and on the 30th, 9,763.

3. The aggregate loss by death from sickness (nine-tenths being from typhus) in the French hospitals on the Bosphorus exceeded 10,000 during the first quarter of the present year. The daily mortality in twelve of these hospitals in January and February, ranged up to 240.

4. From the 1st of Junuary to the 17th of March, when the transport of typhus cases from the Crimea was discontinued authoritatively, more than 5,000 deaths occurred on board French transports and men-of-war, between the Crimea and the Bosphorus.

5. In the Crimea there were fourteen Field Hospitals, or *Ambulances*, during the same period, each containing from 800 to 1,100 sick. The deaths in each varied from 15 to 20 daily. Thus the aggregate loss by death from disease in these hospitals during this period exceeded 19,000, and is believed to have been very little under 25,000.

6. It is *known* that more than *thirty-four thousand* French soldiers of the Army of the East died from disease during the months of January, February, and March, 1856. It is *believed*

by those able to judge, that those deaths exceeded *forty thousand.*

7. Sixty-four French Surgeons have died in the Crimea and on the Bosphorus since last November. Of 362 Surgeons of all ranks who have served with the French Army since its landing in Gallipoli in the autumn of 1854 to April, 1856, 72 have fallen victims to typhus alone.

8. On the 15th of March, 1856, there were, in the Officers' Hospital at Constantinople, 31 Surgeons in different stages of typhus, and only one combatant officer.

9. Of 840 hospital orderlies and attendants, employed in the sixty days of January and February, 603 were attacked by typhus when on service.—*Med. Times and Gaz.*, June 17, 1856.

